# Rheumatic Valvulopathy in Sub-Saharan Africa: A Cross-Sectional Study of Cameroonian Urban Schools

**DOI:** 10.5334/gh.1414

**Published:** 2025-03-27

**Authors:** Chris Nadège Nganou-Gnindjio, Anicet Gakdang Ladibe, Joël Marie Obama Nyaga, Sandrine Laure Ngambono, Loic Alban Tasong, Jules Thierry Elong, Hursul Geffried Nzongang, Félicité Kamdem, David Chelo

**Affiliations:** 1 Faculty of Medicine and Biomedical Sciences of the University of Yaoundé 1, Cameroon; 2 Faculty of Medicine and Biomedical Sciences, University of Yaoundé 1, Yaoundé, Cameroon; 3 Faculty of Medicine and Pharmaceutical Sciences, University of Douala, Douala, Cameroon

**Keywords:** Rheumatic valvulopathy, children, Yaoundé-Cameroon

## Abstract

**Background:**

Rheumatic heart disease is a post-infectious sequelae of acute rheumatic fever resulting from an abnormal immune response to streptococcal pharyngitis that triggers valvular damage. It is the most commonly acquired heart disease in children, particularly in developing countries.

**Objective:**

This study aimed to describe rheumatic valvulopathy among schoolchildren and adolescents in Yaoundé, Cameroon.

**Methods:**

A cross-sectional echocardiography study was conducted from December 2022 to May 2023 among students aged 5 to 19 in primary and secondary schools in Yaoundé, Cameroon. All students with informed parental consent and who agreed to participate in the study were included. The data collected were entered and analysed using SPSS statistics software version 23.0. The association between the qualitative variables was verified using Odd-Ratio with a 95% confidence interval and a significance level of 5%.

**Results:**

Of the 1020 children recruited, 133 (13.03%) had rheumatic heart disease with a mean age of 11.69 ± 4.09 years. The sex ratio (M/F) was 0.56. Most of the participants lived in urban slums (60.9%). Cardiac murmurs were detected in 23 (17.3%) participants during auscultation, with the majority (78.3%) being located at the mitral focus. The definitive form of rheumatic heart disease was observed in 69 (51.9%) children. Mitral involvement was observed in all participants. Mitral valve abnormalities were primarily characterized by mitral insufficiency in 124 (93.2%) participants, valvular thickening (74.4%), and restriction of movement (42.9%). Age between 10 and 14 years [OR = 2.36; CI = (1.11–5.01)] and residence in urban slums [OR = 2.14; CI = (1.05–4.36)] were significantly associated with an increase in the occurrence of definitive rheumatic valve disease.

**Conclusion:**

Rheumatic heart disease is common among schoolchildren in this setting. It systematically affects the mitral valve. The clinical presentation is usually silent at first.

## Background

Rheumatic fever is a multi-systemic disease resulting from an autoimmune reaction to group A beta-hemolytic streptococcal pharyngitis in genetically predisposed individuals (1). Its major complication is rheumatic heart disease (RHD) (2). It causes inflammation of the heart valves, leading initially to clinically silent valvulopathy and eventually, to severe and permanent damage leading to heart failure (1). Rheumatic valvulopathy is the most commonly acquired heart disease worldwide (3). According to the most recent estimates of the global burden of disease, it affects around 40.5 million people (4), and approximately 80 million people worldwide may be asymptomatic. The disease is responsible for about 275,000 deaths a year (5). Its prevalence has been steadily declining in developed countries for several decades thanks to improved socio-economic conditions (2). It has been virtually eradicated in Europe and North America, with a few sporadic cases, most of them imported (6). In sub-Saharan Africa, several recent screening surveys on the community burden of disease have been published, and the results show that rheumatic valvulopathy occurs in 1–3% of school-aged children aged between 5 and17 years in the region (5). In Cameroon, however, its actual prevalence in the general population has not been established. However, a hospital-based study at Shisong-Bamenda (a rural setting) between 2005 and 2007 found that out of 262 children recruited with heart murmurs, 169 (64.5%) were diagnosed with rheumatic valvulopathy (7). At the Yaoundé General Hospital, a study was conducted on the registry of the cardiac echography unit for the years 2003–2013. Of 1130 initial echocardiographic examinations performed in children aged ≤18 years, 65 (5.8%) were echocardiographically diagnosed with definitive rheumatic valvulopathy (8).

Although clinical assessment has been used to diagnose the disease for decades, it lacks the sensitivity to detect most cases (9,10). Recent studies show that echocardiographic-based screening improves the detection of rheumatic valve disease (RVD). For a more accurate diagnosis, transthoracic cardiac Doppler ultrasound can be used using the 2012 World Heart Federation (WHF) criteria, which are more sensitive in the early detection of RVD (11). However, the reported clinical and echocardiographic features are limited to a few countries. These largely preventable valvulopathies are grafted with significant morbidity and mortality from complications such as congestive heart failure and arrhythmias, including atrial fibrillation, stroke, infective endocarditis, etc. (9,12,13). Despite the importance of the problem, data on RVD in the general population, particularly in young children and adults, are scarce in Cameroon. The aim of this study was to describe the clinical and echocardiographic aspects of rheumatic valvulopathy in schoolchildren and adolescents in Yaoundé, Cameroon.

## Methods

### Study Design and Setting

A cross-sectional study was conducted with prospective data collection in six schools in the city of Yaoundé, Cameroon. These comprised three primary schools:

- Primary school of the centre, located in Mfoundi department, Yaounde III arrondissement;
- Solidarity Bilingual School Complex primary school, situated in Mefou and Afamba department, Nkolafamba district;
- Leaders Educational Center (LEDUC) in the Mefou department and Akono in Etoa (Mefou-Assi).

And three secondary schools:

- Saint Stephen’s internationnal college located in the Mfoundi department, Yaoundé III district, in Nsimeyong III (Essono city);
- Solidarity Bilingual School Complex College, located in Mefou and Afamba department, Nkolafamba district;
- Nkolbiyen High School in Mefou and Akono department, Mbankomo district.

The study lasted six months, from December 2022 to May 2023, with a four-month data collection period from February 2023 to May 2023.

### Study population

The source population was schoolchildren and adolescents in the city of Yaoundé. All children and adolescents aged between 5 and 19 who had received parental consent and agreed to participate in the study were included.

### Sample size estimation

A four-stage sampling was conducted, covering six schools in Yaoundé, Cameroon, and one primary and secondary school per selected district. The final units chosen randomly were classrooms.

The minimum size for each school was estimated using the following formula applicable to cross-sectional studies: \[
\boldsymbol{n = \frac{\delta * Z^{2} * P * Q} {e^{2}}}
\].

The sample size was estimated to be **169 participants per school selected**. The prevalence of RVD in schools was taken from a meta-analysis of studies from East Africa, i.e., 1.79% (14).

### Data collection

Once in the selected schools, the teachers and children were informed about the study and the various procedures. Each child was then given a letter in an envelope. It contained an information leaflet, an informed consent form and a socio-demographic and economic data questionnaire. These documents had to be completed by the parents or legal guardians. The forms were returned the following day. Students who returned completed forms with written consent were recruited for the study. They then underwent a complete clinical examination and cardiac echography. Data were collected using a data collection sheet. For each participant, we collected socio-demographic, clinical and echocardiographic data.

### Definition of operational terms

The 2012 World Heart Federation’s modified criteria (11) to define rheumatic valvulopathy in people ≤20 years of age were used as previously described.

- Definitive RVD was considered present when there was one of the following echocardiogram findings: (1) pathological mitral regurgitation and at least two morphological rheumatic valvulopathy features of the mitral valve; (2) pathological aortic regurgitation and at least two morphological rheumatic valvulopathy features of the aortic valve; or (3) borderline disease of the aortic and mitral valves.
- Borderline RVD was considered to be present when one of the following was observed: (1) at least two morphological features of the mitral valve without pathological mitral regurgitation or mitral stenosis; (2) the presence of pathological mitral regurgitation; or (3) the presence of pathological aortic regurgitation.

Using the same criteria, pathological mitral regurgitation was defined as mitral regurgitation observed in two echocardiographic views and at least one view. The jet length is ≥2 cm, while pathological aortic regurgitation was defined as aortic regurgitation observed in two views and at least one view; the jet length is ≥1 cm.

## Statistical analysis

The data collected were recorded and then analysed using SPSS version 23.0. Tables and figures were drawn up using Microsoft Office Excel 2016. Categorical variables were expressed as numbers and proportions. Quantitative variables were expressed as the mean with the standard deviation. The association between categorical variables was checked using the Odd-Ratio with a 95% confidence interval and a significance level of 5%.

## Results

### Prevalence of rheumatic valvulopathy

**Figure 1:**
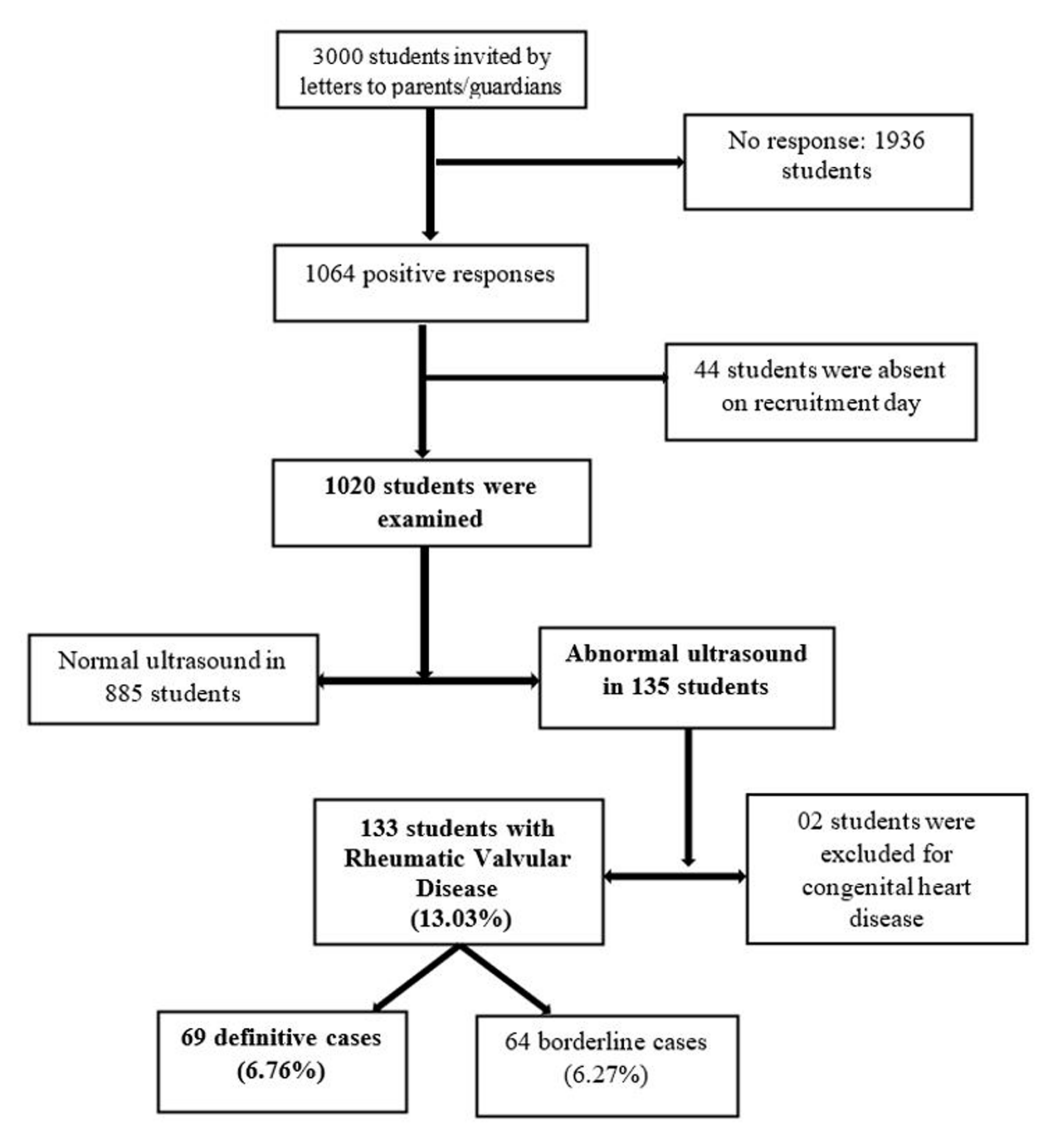
Participant flow chart.

Three thousand students were invited by letters addressed to parents/legal guardians. Of these, there was no response from 1936 students. Out of the 1064 students whose parents/legal guardians gave the authorisation to participate in the study, 44 students were absent on the day of recruitment. Ultimately 1020 participants were recruited. Of the 1020 pupils examined, 388/1020 (38%) were aged between 10 and 15, 540/1020 (52.9%) were at primary level, and 561/1020 (55%) were females. Out of the 1020 students examined, 133 were recorded with rheumatic valvulopathy, giving an overall prevalence of 13.03%, as shown in Figure 1. Considering each echocardiographic form, 69 definite and 64 borderline forms offered a prevalence of 6.76% and 6.27% respectively.

### Socio-demographic and economic characteristics

The mean age of students with RVD was 11.69 ± 4.09 years, with extremes of 5 and 19 years. It was 11.80 ± 3.91 years for definitive valve disease and 11.58 ± 4.29 years for borderline valve disease. Table 1 shows that the sex ratio (M/F) was 0.56; that participants lived with both parents (76.7%), and in urban slums (60.9%). Table 2 shows that there were 50 pupils per classroom (63.9%) and that the distance between home and school was 1 to 5 km (62.5%). The majority of pupils walked to school (66.9%). Table 3 shows that the average age of the head of household was 45.29 ± 7.25 years. Most parents/guardians were between 40 and 50 years old (79.7%) and worked in the informal sector (54.1%). The monthly household income in CFA francs was between 100,000 and 150,000 in 59.4% of cases (1 CFA franc ≈ 0.0016 US dollars). Most mothers had a higher education (57.9%). Where children lived with a rheumatic valvulopathy, 64.7% of households had two to three persons per bedroom. The household flooring was mainly tiled (55.6%). The majority of households (41.4%) used the wall tap as their source of tap water. *See* Tables 4 and 5.

**Table 1:** Socio-demographic characteristics of students who had RVD.


VARIABLES	VALUES (N = 133)	PERCENTAGE (%)

**Age range in years**		

[5–10]	48	36.1

[10–15]	44	33.1

[15–20]	41	30.8

**Gender**		

Male	48	36.1

Female	85	63.9

**Ethnicity**		

Bantu	74	55.6

Semi-Bantu	52	39.1

Sudanese	7	5.3

**Household type**		

Single-parent	31	23.3

Two-parent	102	76.7

**Residence**		

Urban	81	60.9

Semi-Urban	28	21.1

Rural	24	18.0


**Table 2:** Environmental characteristics of students with RVD according to school.


VARIABLES	VALUES (N = 133)	PERCENTAGE (%)

**Level of school**		

Primary	73	54,9

College	60	45,1

**Area of classroom (m^2^)**		

<50	71	53,4

≥50	62	46,6

**Students per classroom**		

≤50	85	63,9

[50–100]	48	36,1

**Distance from home to school (km)**		

<1	19	14,3

[1–5]	83	62,4

[5–10]	21	15,8

≥10	10	7,5

**Type of transport used from home to school**		

None (walking)	89	66,9

Motorcycle	14	10,5

Car	30	22,6


**Table 3:** Socioeconomic and professional status of parents/legal guardians of students with RVD.


VARIABLES	VALUES (N = 133)	PERCENTAGE (%)

**Age range in years**		

[20–30]	1	0.8

[30–40]	20	15.0

[40–50]	106	79.7

≥60	6	4.5

**Professional status**		

Public sector employee	41	30.8

Private sector employee	14	10.5

Informal sector worker	72	54.1

Unemployed	4	3.0

Retired	1	0.8

Others	1	0.8

**Mother’s scholar level**		

Primary	13	9.8

Secondary	42	31.6

University	77	57.9

Non-schooling	1	0.7

**Monthly household income (F CFA)**		

<50.000	10	7.5

[50.000–100.000]	18	13.5

[100.000–150.000]	79	59.4

[150.000–200.000]	5	3.8

≥200.000	21	15.8


1 franc of CFA ≈ 0.0016 US Dollars.

**Table 4:** Type of residence of the students with RVD.


VARIABLES	VALUES (N = 133)	PERCENTAGE (%)

**Flooring materials in house rooms**		

Soil/sand	4	3.0

Tile	74	55.6

Cement	53	39.9

Others	2	1.5

**Number of people sharing the same house**		

[0–2] person	9	6.8

[2–3] persons	86	**64.7**

[3–5] persons	32	**24.0**

>5 persons	6	**4.5**

**Number of persons sharing the same bedroom in the house**		

1	17	12.8

2	55	**41.4**

3	28	**21.0**

4	23	**17.3**

5	8	**6.0**

6	2	**1.5**


**Table 5:** Source of drinking water for households.


VARIABLES	VALUES (N = 133)	PERCENTAGE (%)

**Improved Source**		

None	1	0.8

Household tap	55	41.4

Public tap /fountain	20	15.0

Pump /drilling wells	39	29.3

Protected shaft	13	9.7

Mineral water	5	3.8

**None Improved Source**		

None	108	81.2

Non Protected shaft	10	7.5

Unprotected source of water	11	8.3

Surface water (marsh. river. etc.)	4	3.0


### Clinical characteristics

A history of sore throat was present in 89 (66.9%) participants and fever in 98 (73.7%) participants in the previous 12 months. In the study population, as shown in Table 6, most students (94.7%) had no symptoms. Chest pain was present in 6 (4.5%) participants. Most participants were in good nutritional condition (69.2%). A murmur was present in 23 (17.3%) of pupils. Murmur was present at the mitral focus in 18 (78.3%) students, systolic in 22 (95.7%) students and intensity 2/6 in 21 (91.3%) students. No radiating murmurs were found.

**Table 6:** Distribution of students with RVD according to clinical signs.


VARIABLES	VALUES (N = 133)	PERCENTAGE (%)

**Symptoms**		

None	126	94.7

Chest pain	6	4.5

Dyspnea	1	0.8

**Nutritional status**		

Normal	92	69.2

Underweight	28	21.1

Overweight	12	9.0

Obese	1	0.7

**Cardiac auscultation**		

Normal	108	81.2

Murmur	23	17.3

Arrhythmia	2	1.5


### Echocardiographic findings

**Figure 2:**
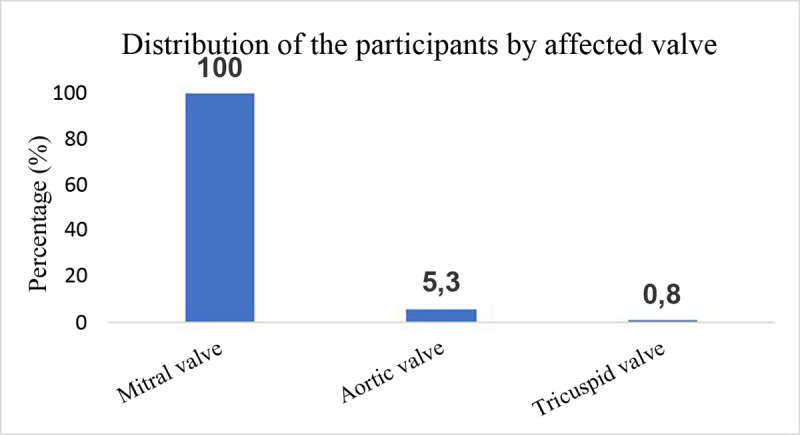
Distribution of participants by affected valve.

As shown in Figure 2, all positive students had mitral valve involvement (100%), the aortic valve was involved in 7 (5.3%) students and the tricuspid valve in 1 (0.8%) student.

Table 7 shows that morphological damage to the mitral valve was predominant in 99 (74.4%) cases, mainly represented by valvular and/or chordal thickening in 99 (74.4%) cases and restriction of valvular motion in 57 (42.9%) cases. Aortic thickening was present in two (1.5%) students.

**Table 7:** Distribution of students with RVD according to morphological characteristics and lesions of affected valves.


VARIABLES	VALUES (N = 133)	PERCENTAGE (%)

**Mitral valve**		

Normal	34	25.6

Abnormal	99	74.4

Valvular and/or cord thickening		

Yes	99	74.4

No	34	25.6

Movement restrictions		

Yes	57	42.9

No	76	57.1

Prolapse		

Yes	7	5.3

No	126	94.7

Calcifications		

Yes	1	0.8

No	132	99.2

**Aortic valve**		

Normal	131	98.5

Irregular or focal thickening	2	1.5

**Monovalvular**		

Mitral insufficiency	124	93.2

Mitral stenosis	1	0.8

Mitral disease	1	0.8

**Bivalvular**		

Mitral insufficiency and aortic insufficiency	6	4.4

**Polyvalvular**		

Mitral insufficiency + aortic insufficiency + tricuspid insufficiency	1	0.8


In Table 7, univalvular lesions were essentially dominated by mitral insufficiency, with 124 (93.2%) cases. Bivalvular lesions combined with mitral and aortic insufficiency were present in 6 (4.4%) participants. Polyvalvulopathy was present in 1 student (0.8%).

Age between 10 and 14 years [OR = 2.36; CI = (1.11–5.01)] and residence in urban slums [OR = 2.14; CI = (1.05–4.36)] were significantly associated with an increase in the occurrence of definitive RVD.

## Discussion

This research aims to contribute to finding solutions to the difficulties frequently encountered in the screening and diagnosis of rheumatic valvulopathies in sub-Saharan African populations in general and those of Cameroon in particular.

The 2012 World Heart Federation echocardiographic criteria standardized RHD identification and improved early case detection. The 2023 guidelines now introduce new screening and confirmatory echocardiographic criteria for RHD. If the 2023 guidelines were used, the RHD of the study population would be classified based on stages and the risk of progression to more advanced valvular heart disease.

This study’s overall prevalence of rheumatic valvulopathy was 13.03% out of 1020 students recruited (Figure 1). There were 69 definitive cases (6.76%) and 64 borderline cases (6.27%), which is a higher incidence than has been reported in other parts of Africa (15). This variation is explained by differences in exposure to streptococcal infection and access to healthcare (16,17). Furthermore, these studies considered only definitive valvulopathy.

The mean age of the pupils was 11.69 ± 4.08 years, with 33.1% of students between 10 and 15 years old having valvular rheumatic disease (Table 1). These results are similar to the average age of 11.64 ± 2.38 years, as reported by Ekure et *al*. in Nigeria in 2017 (15). It can be estimated that by these ages, children with a genetic predisposition to rheumatic fever have already had several episodes of the disease, with a greater probability of valvular involvement. In this study, the number of girls with RVD was higher than that of boys, with a sex ratio (M/F) of 0.56. Although this figure was not reported for the general population, in the population that had RVD, 63.9% were female (Table 1). It should be noted that this is a trend reported in the literature (18). The predominance of rheumatic valvulopathy in women was reported by Marijon et *al*. in Mozambique in 2007 (19), where the difference with boys was significant. The reasons for the increase in rheumatic valvulopathy in women are not well understood (20,21,22), although it is generally known that most autoimmune diseases affect women more than men (23). However, a recent study has shed light on possible reasons for the female predominance in RHD, implicating prothymosin alpha as a potential regulator of sexual predisposition in the disease (24).

The majority of students (60.9%) in this study lived in urban slums in the city of Yaoundé. This result is comparable to that reported by Ngaïdé et *al*. (25) in Senegal in 2015, where children aged over 14 living in the suburbs of Dakar were the most affected. This result could be explained by the high population density. Rheumatic fever and, consequently, rheumatic carditis are diseases of poverty (26).

Most of the students (66.9%) with rheumatic valvulopathy in our study had a history of sore throat. This observation highlights the high prevalence of sore throat in children in this context as a factor linked to the occurrence of rheumatic valvulopathy. The results of this study differ from those observed by Kimbally-Kaky et *al*. (27) in Brazzaville (Republic of Congo), where all students with rheumatic valvulopathy had a history of angina. The fact that our result was lower could be explained by possible memory bias and by the fact that the history-taking in our study was limited to the last 12 months. In our research, most students (94.7%) had no complaints at diagnosis, maybe because valvular lesions are only in their early stages and cannot impact cardiac function. Among those with complaints, the most common symptom was chest pain in 6 (4.5%) students. It was not statistically significant for definitive valve damage. It is possible that these chest pains had origins other than RHD, as no pericardial damage was found on ultrasound in any of the affected students. Underweight was present as a factor in 21.1% of the students in the study. However, there was no association with the degree of valve damage. This is similar to the findings of Kazahura et *al*. (28) in 2019 in Tanzania, where 25% of pupils with RVD were underweight. Although studies have reported an association between rheumatic fever and low body weight, most children in our study had normal nutritional status. In this study, 108 pupils (81.2%) had a typical cardiac auscultation result. Heart murmur was the most common clinical presentation on auscultation in 23 pupils (17.3%). This murmur was predominantly located at the mitral focus (78.3%), confirming the low sensitivity of cardiac auscultation in the detection of RVD (20). However, the sensitivity of cardiac auscultation in our study was high compared with that reported by Marijon et *al*. in Mozambique in 2007 (10%) (20); by Godown et *al*. in 2015 in Uganda (16.4%) (29); and by Chillo et *al*. in 2019 in Tanzania (6.3%) (5). This may be explained by the fact that cardiac auscultation was performed by final year medical students in their studies. All participants had mitral valve involvement (100%). The aortic and tricuspid valves were involved in 5.3% and 0.8% of cases, respectively (Figure 2). Typical morphological features of mitral valve disease observed in this study were valvular and/or chordal thickening in 74.4% of participants, and restriction of movement in 42.9% of cases. Aortic valve thickening was observed in 2 (1.5%) students. In the study, morphological aspects of the mitral valve, such as restriction of motion, valvular prolapse and aortic valve damage, were associated with definitive damage. This could be explained by the pathophysiological phenomena of inflammation in the natural course of RVD, as it is a long-term phenomenon. Inflammation first leads to thickening of the valve and/or chords, followed by restriction of movement and prolapse if nothing is done. The difference in the number of lesion morphologies shows that most of our participants are only at the beginning of these phenomena. If nothing is done, it will lead to definitive mutilation of the valve responsible for heart failure. Our results are similar to those reported by Chillo et *al*. in 2019 in Tanzania (5), where out of 95 students with RVD, 56 (58.9%) had valvular or subvalvular thickening, and 45 (47.3%) had mitral valve deformation, while aortic valve thickening was detected in only 2 students (2.1%). Single-valve lesions were most frequent in 126 cases (94.73%), and all of these lesions concerned the mitral valve. Bivalvular lesions were present in 6 patients (4.4%), and all of them were mitro-aortic. Tri-valvular involvement was observed in 1 patient (0.8%). These results are similar to those of Marijon et *al*. in Cambodia in 2007 (19), who found 77.2% pure mitral damage and 10.12% mitro-aortic damage. The mitral valve is frequently involved in rheumatic valvulopathy (30), probably because the mitral valve cusps are exposed to left ventricular pressure during systolic contraction. However, the aortic cusps are exposed to aortic diastolic pressure during closure, so the shear stress on the large leaflet of the mitral valve is higher than on the small aortic cusps, making the mitral valve more prone to injury during attacks of rheumatic fever (28).

### Limitations

This study utilized transthoracic echocardiography to screen a large sample of schoolchildren, providing data on rheumatic valvulopathy focused on a vulnerable population in sub-Saharan Africa, particularly in Cameroon. The use of echocardiography allows for identifying both clinically apparent and subclinical cases of RHD. These results contribute to the global understanding of RHD, particularly in low-resource settings, and can inform international efforts to combat the disease.

However, several limitations of this study should be noted: the small sample size limited the analysis possible for an observational study performed in a single urban setting with its subjects selected by convenience; the unavailability of some information (such as distance from home to health center, prophylaxis against rheumatic fever) might have hindered the ability for a better association of clinical and echocardiographic findings in our population; and, as this was an ultrasound assessment, it should be noted that echocardiography shares all of the limitations of an operator-dependent technique. Another limitation of this study is that almost two thirds of children did not respond to invitations. This may be due to the economic hardships of parents, or it may be the result of logistical challenges that make it difficult for their children to participate in studies; it could also be that socio-cultural, education and formal studies may not be prioritized over other activities, such as household chores or agricultural work.

## Conclusion

The prevalence of rheumatic valvulopathy is high among schoolchildren and adolescents living on the periphery of Yaoundé, Cameroon. It systematically affects the mitral valve and is associated with potentially modifiable risk factors. The clinical presentation is usually initially silent, and cardiac auscultation is not very sensitive in detecting these lesions. Children aged between 10 and 14 years living in urban slums and overcrowded households with a history of sore throats represent a high-risk population that should be targeted for early detection and management, as well as rational prophylaxis. It is therefore important to emphasize an extensive screening of this population group. Early detection in this population can lead to timely interventions and to better long-term outcomes.

## Data Accessibility Statement

The datasets used and/or analysed during the current study are available from the corresponding author upon reasonable request.

## Abbreviations

AI: Aortic insufficiency

MI: Mitral insufficiency

MS: Mitral stenosis

RHD: Rheumatic heart disease

RVD: rheumatic valve disease

TI: tricuspid insufficiency

VD: valvular disease

WHF: World Heart Federation
